# A Case of Aseptic Meningitis Presenting With Widespread Non‐Dermatomal Neuropathic Pain

**DOI:** 10.1002/jgf2.70107

**Published:** 2026-02-23

**Authors:** Jun Komoda

**Affiliations:** ^1^ Department of Internal Medicine Atsumi Hospital Aichi Japan

**Keywords:** aseptic meningitis, case report, differential diagnosis, inflammatory cytokines, neuropathic pain, non‐dermatomal

## Abstract

Aseptic meningitis occasionally causes neuropathic pain in a dermatomal distribution, but widespread non‐dermatomal pain is extremely rare. We report a case of a 62‐year‐old man with ulcerative colitis who developed neuropathic pain involving the head, trunk, and limbs. Cerebrospinal fluid analysis and brain MRI confirmed aseptic meningitis. Although vitamin B12 deficiency and cervical spondylotic myelopathy were present, they did not explain the symptoms. The pain improved with oral analgesics. This case highlights aseptic meningitis as a possible cause of non‐dermatomal neuropathic pain.

## 1 Background

Aseptic meningitis is most commonly caused by viral infection, and is typically self‐limiting [1, 2]. However, when patients do not present with the typical symptoms of meningitis, the diagnosis may be overlooked. We report an extremely rare case of aseptic meningitis presenting with diffuse non‐dermatomal neuropathic pain. To our knowledge, there are no previous reports of patients with aseptic meningitis presenting with non‐dermatomal neuropathic pain.

## 2 Case Presentation

A 62‐year‐old Japanese man with hypertension, ulcerative colitis, chronic sinusitis, and chronic urticaria had been under regular follow‐up at multiple medical institutions. Ulcerative colitis had been diagnosed 7 years prior and remained stable with oral mesalazine without additional immunosuppressive therapy. He had also been receiving long‐term clarithromycin for chronic sinusitis. He had no recent history of travel, vaccination, or new medication use.

**FIGURE 1:**
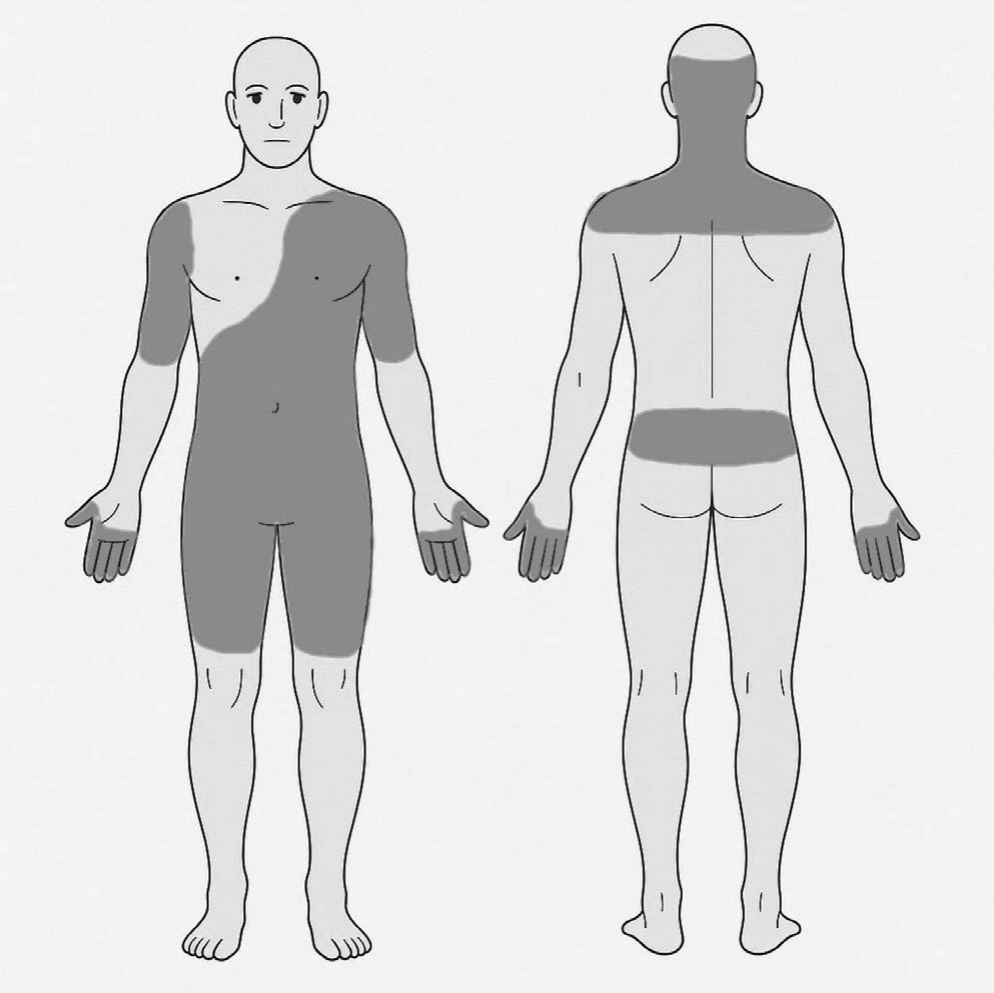
Distribution of pain.

Seventeen days before admission, he reported spontaneous prickling and electric shock–like pain involving the occipital region, posterior neck, bilateral upper arms, left anterior chest, the entire abdomen, lower back, bilateral thighs, and distal fingers (numerical rating scale [NRS] 3), which gradually worsened to NRS 10 (Figure 1). The pain was occasionally triggered by light touch to the skin, consistent with allodynia. Ten days before admission, he developed fever and pharyngeal discomfort; antigen tests for influenza virus and coronavirus were negative. Seven days before admission, despite defervescence, generalized pain persisted, prompting referral to our hospital for further evaluation.

On presentation, vital signs were stable, except for mild tachycardia. Physical examination revealed no abnormalities in the head and neck, pharynx, lungs, heart, abdomen, or joints. No rashes or lymphadenopathy were observed. Chest and abdominal CT revealed no findings that could explain the pain, and he was managed conservatively.

He returned 7 days later because the pain in the head, trunk, and extremities did not improve. He presented with reproducible symptoms and lacked psychiatric manifestations suggestive of psychogenic pain. Neurological examination revealed normal cranial nerves, motor strength, reflexes, and coordination; however, jolt accentuation was positive. Tactile allodynia was present; however, thermal and pain sensation, as well as proprioception, were preserved. Vibration sense was not examined. No skin eruptions were observed. Laboratory test results were largely normal, except for mild hyponatremia (134 mEq/L) and reduced vitamin B12 levels (84 pg/mL). Serological tests for HIV, Epstein–Barr virus, cytomegalovirus, tuberculosis (T‐SPOT), and antineutrophil cytoplasmic antibodies were negative. Cerebrospinal fluid (CSF) analysis revealed 79 cells/μL (94% mononuclear), protein 75 mg/dL, and glucose 55 mg/dL (serum 121 mg/dL; Table 1). Gram staining and bacterial culture results were negative. Brain MRI demonstrated mild meningeal enhancement and an old infarct in the left putamen. Based on these findings, aseptic meningitis was diagnosed.

**TABLE 1:** CSF analysis.

Test	Result	Unit	Reference range
Cell count	79	/μL	< 5
Differential count	94% mononuclear, 6% polymorphonuclear	—	—
Protein	75	mg/dL	15–45
Glucose	55	mg/dL	50–80
ADA	3.2	U/L	< 8.0
IgG index	0.6	—	< 0.7
Oligoclonal bands	Negative	—	Negative
Anti‐aquaporin‐4 antibody	Negative	—	—
Cytology	Negative	—	—
Bacterial/fungal/TB cultures	Negative	—	—
HSV/VZV PCR	Negative	—	—

Abbreviations: ADA, adenosine deaminase; AQP4, anti‐aquaporin‐4 antibody; CSF, cerebrospinal fluid; HSV, herpes simplex virus; IgG, immunoglobulin G; PCR, polymerase chain reaction; TB, tuberculosis; VZV, varicella‐zoster virus.

Empirical ceftriaxone (2 g/day) and acyclovir (700 mg three times daily) were initiated, and clarithromycin was discontinued. Oral amitriptyline (10 mg) was added to control neuropathic pain. Ceftriaxone was discontinued on Day 4, and acyclovir was discontinued on Day 9 after negative CSF culture and herpes simplex virus/varicella zoster virus polymerase chain reaction results. Despite treatment with loxoprofen administered since Day 2, and amitriptyline, the dose of which was increased to 40 mg on Day 8, the pain persisted. Consequently, pregabalin (50 mg daily) was initiated on Day 12, leading to gradual improvement.

On Day 17, the patient reported distal limb numbness, exacerbated by neck flexion. Cervical MRI revealed spinal canal stenosis at C5/6 and a T2 hyperintense lesion consistent with cervical spondylotic myelopathy (Figure S1), which could not explain the diffuse pain or CSF abnormalities. Pain improved to NRS 1 by Day 23, and he was discharged. Two months after discharge, follow‐up CSF analysis demonstrated improvement, with 11 cells/μL (91% mononuclear) and protein 43 mg/dL. At 6 months, distal numbness persisted, but pain in the head and trunk had completely resolved. Oral amitriptyline (40 mg) and pregabalin (50 mg) were continued for residual numbness.

## 3 Discussion

Our case highlights an extremely rare presentation of aseptic meningitis characterized by diffuse, non‐dermatomal neuropathic pain, which contrasts with the more typical dermatomal pain patterns associated with focal central nervous system involvement. The typical symptoms of aseptic meningitis include headache, nausea, and neck stiffness [2]. Although our patient experienced headaches, their features differed from those of typical meningitis‐associated headache, which is generally a migraine‐like nociceptive somatic pain resulting from inflammatory activation of trigeminal sensory afferents [3]. In contrast, the predominant symptom in this case was widespread, electric shock–like pain marked with allodynia, strongly suggesting neuropathic involvement [4]. Differential diagnoses for widespread neuropathic pain include vitamin B12 deficiency, spinal cord disorders, autoimmune or infectious neuropathies, and metabolic diseases. Although our patient had vitamin B12 deficiency and cervical spondylotic myelopathy, neither condition accounted for the clinical course, pain distribution, and meningeal findings. Drug‐induced meningitis was considered; however, it typically develops shortly after exposure, with a reported mean onset of approximately 62 days [5], which was inconsistent with the patient's long‐term medication exposure.

In viral meningitis, inflammatory cytokines such as tumor necrosis factor, interleukin (IL)‐1β, and IL‐6 are produced in the CSF [6, 7]. These cytokines can enhance excitatory synaptic transmission and suppress inhibitory synaptic transmission in the spinal neurons, thereby inducing widespread hyperalgesia and allodynia [8]. Such diffuse sensitization of spinal and supraspinal nociceptive pathways may account for non‐dermatomal pain distribution without focal structural lesions. Although the cytokine levels were not measured, this mechanism may explain the widespread pain observed in this case.

Neuropathic pain associated with central nervous system disorders is typically dermatomal when focal parenchymal lesions directly involve segmental sensory pathways. Previous reports of meningitis caused by *Streptococcus acidominimus* or postherpetic myelitis described dermatomal pain corresponding to localized CNS involvement [9, 10]. In contrast, our patient exhibited widespread, non‐dermatomal pain in the absence of any parenchymal lesions on imaging, suggesting a fundamentally different pain‐generating mechanism, driven by diffuse meningeal inflammation rather than focal neural injury.

## 4 Conclusion

We describe an extremely rare case of aseptic meningitis presenting with diffuse, non‐dermatomal neuropathic pain without typical meningitis‐associated headache or focal neurological deficits. This case underscores the importance of considering aseptic meningitis even in patients lacking classic meningeal symptoms when unexplained widespread neuropathic pain is present.

## Author Contributions

**Jun Komoda:** writing – original draft, writing – review and editing.

## Funding

The author has nothing to report.

## Ethics Statement

The author has nothing to report.

## Consent

Written informed consent was obtained from the patient for the publication of this case report.

## Conflicts of Interest

The author declares no conflicts of interest.

## Supporting information


**Figure S1:** jgf270107‐sup‐0001‐FigureS1.jpg.

## Data Availability

The data that support the findings of this study are available from the corresponding author upon reasonable request.
