# Supplementary figures and images for: A Case of Aseptic Meningitis Presenting With Widespread Non‐Dermatomal Neuropathic Pain
Source: J Gen Fam Med. 2026 Feb 23;27(2):e70107. doi: 10.1002/jgf2.70107 (PMC12929655; doi:10.1002/jgf2.70107)

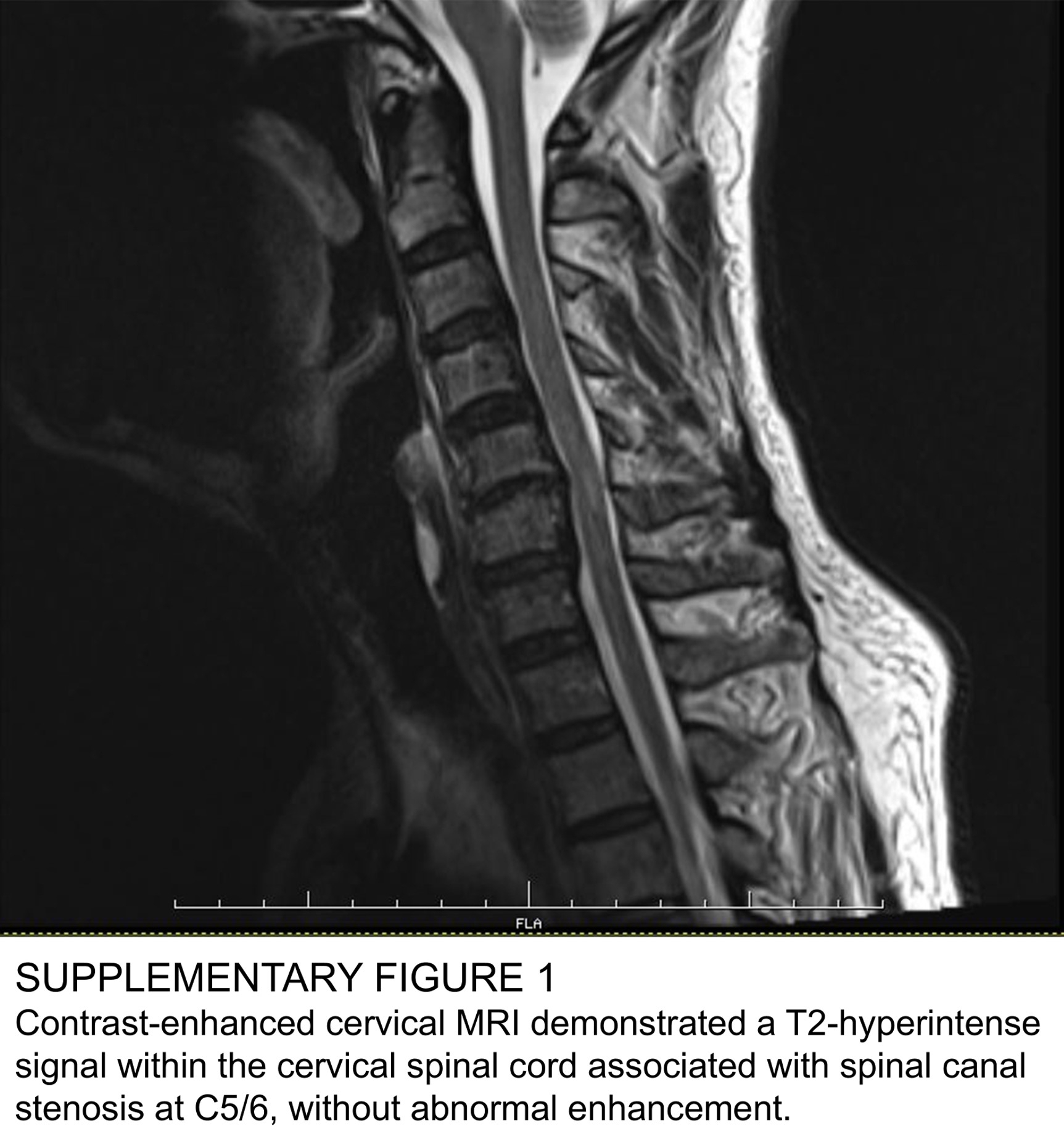

Supplement: Supplementary file 1 — Figure S1: jgf270107‐sup‐0001‐FigureS1.jpg. [file JGF2-27-e70107-s001.jpg]
